# L-theanine prevents progression of nonalcoholic hepatic steatosis by regulating hepatocyte lipid metabolic pathways via the CaMKKβ-AMPK signaling pathway

**DOI:** 10.1186/s12986-022-00664-6

**Published:** 2022-04-15

**Authors:** Juanjuan Liang, Lili Gu, Xianli Liu, Xintong Yan, Xiaowen Bi, Xirui Fan, Jinyi Zhou, Shuai Lu, Lan Luo, Zhimin Yin

**Affiliations:** 1 Jiangsu Province Key Laboratory for Molecular and Medical Biotechnology, College of Life Science, Nanjing Normal University, No. 1 Wenyuan Road, Nanjing, 210046 Jiangsu People’s Republic of China; 2 State Key Laboratory of Pharmaceutical Biotechnology, School of Life Sciences, Nanjing University, Nanjing, 210023 Jiangsu People’s Republic of China

**Keywords:** L-theanine, Hepatic steatosis, Lipid accumulation, CaMKKβ, SREBP-1c

## Abstract

**Background:**

L-theanine, a non-protein amino acid was found principally in the green tea, has been previously shown to exhibit potent anti-obesity property and hepatoprotective effect. Herein, we investigated the effects of L-theanine on alleviating nonalcoholic hepatic steatosis in vitro and in vivo, and explored the underlying molecular mechanism.

**Methods:**

In vitro, HepG2 and AML12 cells were treated with 500 μM oleic acid (OA) or treated with OA accompanied by L-theanine. In vivo, C57BL/6J mice were fed with normal control diet (NCD), high‐fat diet (HFD), or HFD along with L-theanine for 16 weeks. The levels of triglycerides (TG), accumulation of lipid droplets and the expression of genes related to hepatocyte lipid metabolic pathways were detected in vitro and in vivo.

**Results:**

Our data indicated that, in vivo, L-theanine significantly reduced body weight, hepatic steatosis, serum levels of alanine transaminase (ALT), aspartate transaminase (AST), TG and LDL cholesterol (LDL-C) in HFD-induced nonalcoholic fatty liver disease (NAFLD) mice. In vitro, L-theanine also significantly alleviated OA induced hepatocytes steatosis. Mechanic studies showed that L-theanine significantly inhibited the nucleus translocation of sterol regulatory element binding protein 1c (SREBP-1c) through AMPK-mTOR signaling pathway, thereby contributing to the reduction of fatty acid synthesis. We also identified that L-theanine enhanced fatty acid β-oxidation by increasing the expression of peroxisome proliferator–activated receptor α (PPARα) and carnitine palmitoyltransferase-1 A (CPT1A) through AMP-activated protein kinase (AMPK). Furthermore, our study indicated that L-theanine can active AMPK through its upstream kinase Calmodulin-dependent protein kinase kinase-β (CaMKKβ).

**Conclusions:**

Taken together, our findings suggested that L-theanine alleviates nonalcoholic hepatic steatosis by regulating hepatocyte lipid metabolic pathways via the CaMKKβ-AMPK signaling pathway.

**Supplementary Information:**

The online version contains supplementary material available at 10.1186/s12986-022-00664-6.

## Background

Nonalcoholic fatty liver disease (NAFLD) is the most common chronic liver disease, affecting about a quarter of the global population [1]. It is characterized by excess triglycerides (TG) accumulation in liver and defined by the presence of steatosis in > 5% of hepatocytes [2]. The pathological spectrum of NAFLD includes nonalcoholic fatty liver (NAFL) and nonalcoholic steatohepatitis (NASH), according to the severity of disease, NASH includes fibrosis, cirrhosis and hepatocellular carcinoma (HCC) [3]. At present, there are no approved therapies for NAFLD, novel therapeutic approach for NAFLD is urgent needed [4].

In recent years, agents derived from natural sources have been widely studied by researchers due to their low toxicity and various biological effects. L-theanine (γ-glutamylethylamide) is a non-protein amino acid found principally in the tea plant and mushrooms [5]. It shows beneficial effects on various nutritional and metabolic diseases in humans, and it has antioxidative [6], neuroprotective [7, 8], hepatoprotective [9], anti-tumor [10] and anti-restenosis effects [11]. L-theanine can also reduce the risk of the development of diabetes mellitus type 2 (T2DM) [12].

Previous studies have shown that L-theanine can suppress fat accumulation in mice [13], and affect the absorption of lipids by regulating the expression of intestinal fatty acid transporters in rats [14]. L-theanine also can regulate glucose, lipid, and protein metabolism via insulin and AMP-activated protein kinase (AMPK) signaling pathway in normal rats [15]. Recent studies demonstrated that L-theanine can activate the browning of inguinal white adipose tissue through upregulating the expression of thermogenic genes, thus ameliorates diet-induced obesity in mice [16]. L-theanine also can modulate gut microbiota composition, ameliorated adiposity and hepatic steatosis in obesity mice, they also found that L-theanine can regulate the mRNA expression of genes related to lipid metabolic [17]. However, the above studies did not represent the specific mechanisms underlying the effect of L-theanine on nonalcoholic hepatic steatosis, and in the present study, we mainly studied the effects of L-theanine on the simple steatosis of liver.

Sterol regulatory element-binding protein 1c (SREBP-1c), a key lipogenic transcription factor, is synthesized as an inactive precursor (SREBP-1-P) in endoplasmic reticulum (ER), then it cleaved into nuclear form (SREBP-1-N) in the Golgi apparatus. Ultimately, the SREBP-1-N was translocated to the nucleus [18], and activates transcription of genes related to fatty acid synthesis, include fatty acid synthase (FASN) and acetyl CoA carboxylase 1 (ACC1) [19]. Thus, the trafficking, processing, and transcription of SREBP-1c mediates the pathophysiology of nonalcoholic hepatic steatosis.

In the present study, we demonstrated that L-theanine can decrease fatty acid synthesis through inhibiting the nucleus translocation of SREBP-1c via AMPK-mTOR signaling pathway, L-theanine also can promote the fatty acid the β-oxidation through AMPK. Moreover, we found that L-theanine can active Calmodulin-dependent protein kinase kinase-β (CaMKKβ), an upstream kinase of AMPK. Thus, L-theanine ameliorates nonalcoholic hepatic steatosis by regulating hepatocyte lipid metabolic pathways via the CaMKKβ-AMPK signaling pathway, and our study proved that L-theanine may be a novel regent for the treatment of nonalcoholic hepatic steatosis.

## Methods

### Materials and reagents

L-theanine (CAS: 3081-61-6, 98% purity) was purchased from Sigma-Aldrich (St. Louis, MO, USA). Donkey anti-Rabbit IgG H&L (Alexa Fluor 488) (#ab150075; 1:500) was obtained from Abcam (Cambridge, UK). DAPI was purchased from Invitrogen (Carlsbad, CA, USA). STO-609, BAPTA-AM were purchased from Topscience (Shanghai, China).

Primary antibodies against ACC1 (#3676), FASN (#3180), p-AMPK (#2535), AMPKα (#5831) were purchased from Cell Signaling Technology (Beverly, MA, USA). Antibody against p-CaMKKβ (#AF4487), CaMKKβ (#DF4793) were purchased from Affinity Biosciences (OH, USA). Antibody against SREBP-1c (#A15586), phospho-mTOR (#AP0094), mTOR (#A2445), HRP Goat Anti-Rabbit IgG (#AS014), and HRP Goat Anti-Mouse IgG (#AS003) were purchased from ABclonal (Wuhan, Hubei, China). Antibodies against PPARα (#15540-1-AP), CPT1A (#15184-1-AP), SREBP-1c (#14088-1-AP) were purchased from Proteintech (Chicago, IL, USA). Antibodies recognizing GAPDH (#AP0063), Lamin B (#AP6001), and β-ACTIN (#AP0060;) were purchased from Bioworld Technology (Minneapolis, MN, USA). Donkey anti-Rabbit IgG H&L (Alexa Fluor 488) (#ab150075) was obtained from Abcam (Cambridge, UK). DAPI was purchased from Invitrogen (Carlsbad, CA, USA).

### Animals and treatments

Male C57BL/6J mice weighing 20 to 22 g (6 weeks old) were procured from the Beijing Vital River Laboratory Animal Technology Co. (Beijing, China; SCXK 2019–0001). All animals were kept under standard conditions with a controlled temperature and a 12-h light/dark cycle with water and food ad libitum. After an acclimatization period of 1 week, all mice were randomly divided into 3 groups (n = 8/per group). The normal control diet (NCD) group, the high-fat diet (HFD) group (60% fat, D12492; Research Diets, New Brunswick, NJ, USA), and the L-theanine group (fed with HFD supplement with 300 mg/kg L-theanine). The NCD, HFD, and L-theanine group were fed for 16 weeks and in the meanwhile treated with saline or 300 mg/kg L-theanine by gavage once daily for 16 weeks. Food intake and body weight was recorded weekly. At the end of the experiment, all mice have fasted for 16 h before sacrifice, then blood samples, liver and visceral adipose tissues were harvested, weighed, and stored at − 80 °C, Additional sections of liver and visceral adipose tissues were prepared for histological analyses.

All treatments of mice in this study were in strict agreement with the “Guide for the Care and Use of Laboratory Animals”. All the experimental procedures were approved by the Animal Ethics Committee of Nanjing Normal University.

### Biochemical analysis

The blood samples were collected from orbital vascular plexus and centrifuged at 3000 rpm for 10 min, then serum was collected for biochemical analysis. Serum levels of alanine aminotransferase (ALT), aspartate aminotransferase (AST), HDL cholesterol (HDL-C), LDL cholesterol (LDL-C), total triglycerides (TG) and total cholesterol (TC), were detected using commercial assay kits according to the manufacturer's instructions (Nanjing Jiancheng Bioengineering Institute, Nanjing, China).

TG and TC contents in mouse liver and hepatocytes were also measured using commercial kit (Nanjing Jiancheng Bioengineering Institute, Nanjing, China) according to the manufacturer’s protocol.

### Oil red O staining

Liver tissues were fixed with 4% polyformaldehyde and embedded in tissue freezing medium (Leica). The liver sections (8 μm) and treated hepatocytes were immersed in Oil Red O working solution (Sigma-Aldrich) for 20–30 min at room temperature, then washed with 60% isopropanol to remove unbound dye and counterstained with hematoxylin. Finally, images were captured by using a light microscope (Nikon, Japan).

### Histology and Immunohistochemistry

Liver and adipose tissues were fixed in 4% paraformaldehyde and embedded in paraffin. Sections (4 μm) were deparaffinized with xylene and rehydrated in graded ethanol, stained with hematoxylin and eosin (H&E). The images were taken using a microscope (Nikon, Japan), and the NAFLD score was evaluated as previously described [20].

For immunohistochemistry, paraffin sections of liver were deparaffinized and rehydrated, treated for antigen retrieval, and endogenous peroxidase was quenched with 3% hydrogen peroxide. After blocking nonspecific antigen, the slides were then incubated with primary antibody overnight at 4 °C, followed by biotinylated secondary antibody and third antibody coupled with SABC (BosterTech, China). The DAB kit was used to produce a brown stain, and counterstained with hematoxylin. Images were acquired by using a light microscope (Nikon, Japan).

### Cell culture and treatments

The HepG2 and AML12 cells were purchased from Cell Bank of the Chinese Academic of Sciences (CBCAS), Shanghai, China. HepG2 cells were cultured in EMEM (Wisent, Canada), supplemented with 10% fetal bovine serum (Wisent, Canada), 100 U/mL penicillin and 100 μg/mL streptomycin (Wisent). AML12 cells were cultured in DMEM/F12 (Wisent, Canada) supplemented with 10% fetal bovine serum, 100 U/mL penicillin, 100 μg/mL streptomycin, ITS supplement and 40 ng/mL dexamethasone. All cells were cultured in humidified atmosphere containing 5% CO2 at 37 °C (Thermo Fisher Scientific).

20 mM OA solution was obtained by dissolving OA in 0.1 M NaOH at 75 °C in a water bath, then added to 20% BSA, filtered using 0.22 μm filter, a final stock solution of 10 mM OA were prepared. The 10% BSA solution was added as a control.

### Cell viability assay

Cell viability was determined using cell counting kit-8 (CCK-8). according to the manufacturer's instructions (Vazyme, Nanjing, China).

### Immunofluorescence microscopy

HepG2 cells grown on coverslips were pretreated with or without L-theanine for 2 h, after co-incubated with 500 μM oleic acid (OA) for 24 h, The cells were washed with PBS and fixed with 4% formaldehyde for 15 min at room temperature, then cells were permeabilized in 0.2% TritonX-100 for 20 min followed by blocking with 5% BSA for 30 min. After that, the cells were incubated anti-SREBP-1c antibody (1:100) overnight at 4 °C and incubated with Donkey anti-Rabbit IgG H&L (Alexa Fluor 488) secondary antibody for 1 h. Cell nuclei were stained with DAPI for 5 min. Images were captured by the Nikon A1 microscope (Tokyo, Japan).

### Real-time PCR

Total RNA from livers was extracted using RNA Isolation Kit (Omega Bio-tek, America) and then was reversely transcribed to cDNA using PrimeScript RT reagent kit (TaKaRa, Shiga, Japan). Quantitative polymerase chain reaction (qPCR) was carried out on ABI StepOnePlus real‐time PCR system (Applied Biosystems, USA) using SYBR Premix Ex Taq (TransGen Biotech, China). The results were analysed using the 2^−ΔΔCt^method. Values were normalized to β‐actin. The sequences of primers were listed in the Additional file 1: Table S1.

### Preparation of cytoplasmic and nuclear extracts

Nuclear and cytoplasmic extracts were lysed with NE-PER Nuclear and Cytoplasmic Extraction Reagents (Thermo Fisher Scientific, Rockford, IL, USA) according to the manufacturer’s instructions.

### Immunoblotting

Liver samples or cells were lysed in RIPA lysis buffers (Beyotime, China) supplemented with protease inhibitor cocktail and phosphatase inhibitors. A total of 20–50 μg lysates were loaded onto SDS-PAGE gels and transferred to a PVDF membrane (Millipore, China). After blocking with TBS/T (0.1% Tween-20) containing 5% nonfat dry milk for 1 h at room temperature, the membranes were incubated with primary antibody at 4 °C overnight followed by HRP-conjugated secondary antibody. Immunoreactive proteins were visualized using the ECL immunoblotting system from Tanon (Shanghai, China).

### Measurement of intracellular Ca^2+^

Fluo-4 AM Ca^2+^ probe (Beyotime, China) was used for measuring the intracellular Ca^2+^ concentration according to manufacturer’s instruction. In brief, after drug treatment, cells in 6-well plates were washed three times with PBS and incubated with Fluo-4 AM (4 μM final concentration) in PBS at 37 ℃ in a 5% CO_2_ containing incubator for 40 min. The cells were then washed three times again with PBS and incubated for another 20 min in PBS containing 1% FBS to ensure that Fluo-4 AM was completely converted into Fluo-4. Cells were then imaged by inverted fluorescence microscope (Lecia, Germany). Intracellular Ca^2+^ fluorescence value was detected as previously study described [21], briefly, cells stained with Fluo-4 AM in 6-well plates were digested with 0.25% trypsin without EDTA and washed with PBS, followed by dilution to 2 × 10^6^ cells/ml with PBS. Finally, cell suspension was added into a black clear bottom 96-well plate (150 μl/well), and the fluorescence value was measured by multifunctional microplate reader under the condition of 488 nm excitation wavelength and 520 nm emission wavelength.

### Statistical analysis

All data were analyzed using GraphPad Prism 6.0 (GraphPad Software, San Diego, CA). All experimental results are presented as mean ± SEM. Statistical analysis between two groups were determined using the two-tailed Student’s *t*-test. One-way ANOVA followed by the Tukey multiple comparisons test was used for comparison between multiple groups. A *P* value of less than 0.05 was considered to be statistically significant.

## Results

### L-theanine alleviates hepatic steatosis in OA induced HepG2 and AML12 cells

To elucidate the effects of L-theanine on free fatty acid-induced hepatic steatosis, an in vitro model of nonalcoholic hepatic steatosis was established by stimulating HepG2 and AML12 cells with OA for 24 h according to previous studies [22, 23]. As retain some characteristics of liver cells [24], HepG2 cells are a suitable model to study the lipid metabolism of liver. However, such carcinoma derived cell lines have genetic and characteristic limitations [25]. To overcome its potential shortcomings, we also used mouse hepatocyte cell line AML12 to rigorously demonstrate the effects of L-theanine on hepatic steatosis.

**Fig. 1:**
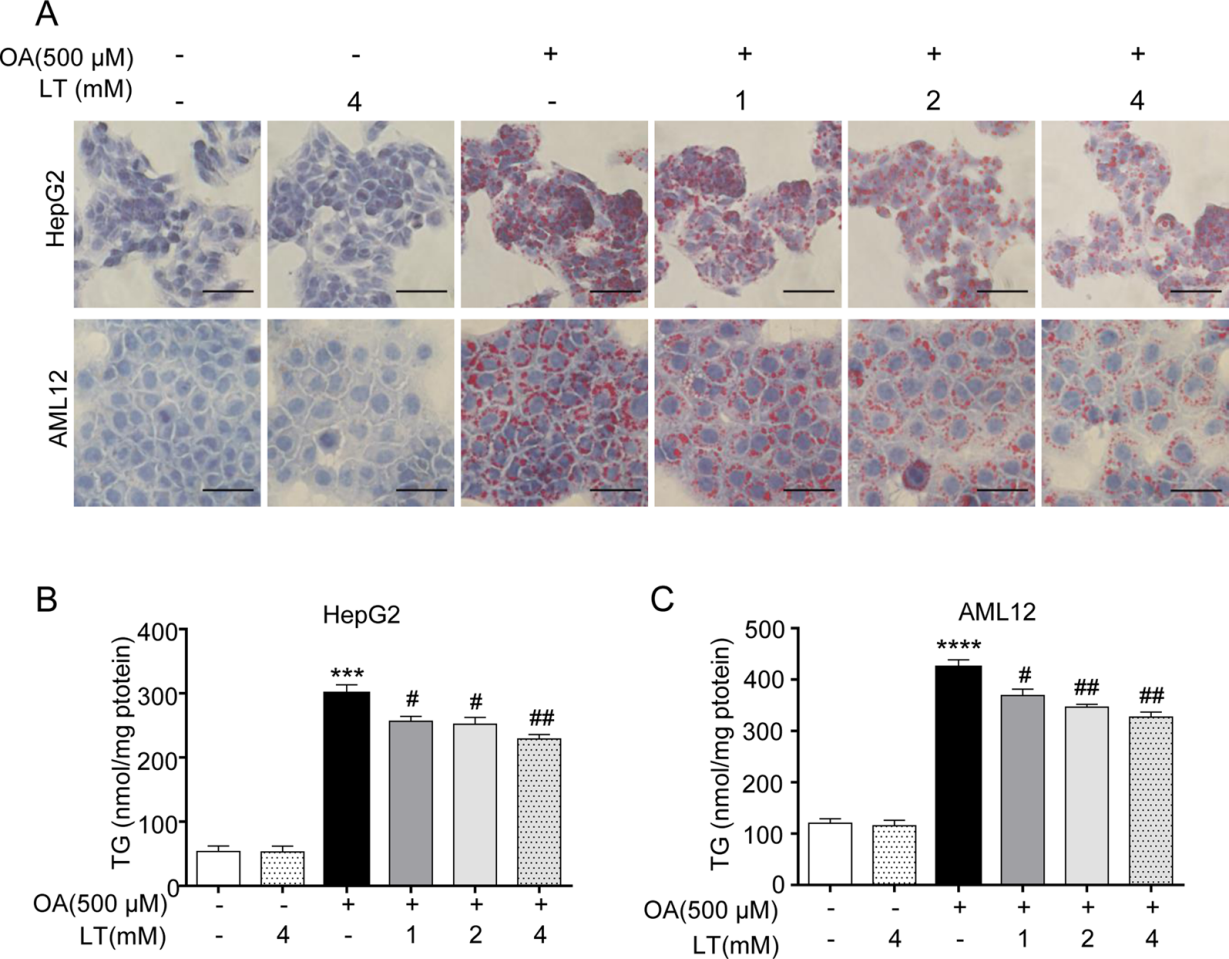
L-theanine alleviates hepatic steatosis in OA induced HepG2 and AML12 cells. HepG2 and AML12 cells were treated with 500 μM OA for 24 h with or without pretreated L-theanine for 2 h. **A** Representative Oil Red O staining of HepG2 and AML12 cells. Scale bar: 50 μm (40 ×). Intracellular TG levels in HepG2 (**B**) and AML12 (**C**) cells. Values are expressed as mean ± SEM of three independent experiments. ***p < 0.001, ****p < 0.0001 versus control group; ^#^*p* < 0.05, ^##^*p* < 0.01 versus OA group. LT, L-theanine

First, cytotoxicity experiment revealed that L-theanine had no effect on cell viability at the concentration of 1–4 mM (Additional file 2: Fig. S1A, B). At the concentration of 0–500 μM, OA also had no effect on cell viability (Additional file 2: Fig. S1C, D). So, we chose the concentrations of L-theanine (1–4 mM) and OA (500 μM) for subsequent experiments. Next, HepG2 and AML12 cells were treated with 500 μM OA for 24 h with or without pretreated L-theanine for 2 h, Oil Red O staining reveled that lipid droplets were markedly increased in OA group, while L-theanine significantly suppressed intracellular lipid accumulation in a dose-dependent manner (Fig. 1A). Intracellular levels of TG in HepG2 (Fig. 1B) and AML12 (Fig. 1C) cells were also remarkably decreased by L-theanine compared with OA group. These results above suggested that L-theanine attenuated steatosis in hepatocyte through decreasing the TG content.

### The effects of L-theanine on the metabolism of normal mice

At first, we explored the effect of L-theanine on mice fed normal control diet (NCD). Mice were randomly divided into four groups (n = 6), gavaged with 150, 300, 600 mg/kg L-theanine for 16 weeks once daily, saline were gavaged as control. The concentration of L-theanine given to mice was based on previous study[9]. As the result showed, L-theanine had no significant effects on body weight (Additional file 3: Fig. S2A, B) and the serum levels of ALT, AST, TG, TC, LDL-C (Additional file 3: Fig. S2C). However, L-theanine significantly increased the serum level of HDL-C (Additional file 3: Fig. S2C), and there was a trend that L-theanine decreased the levels of ALT, AST, TG in serum. Above data indicated the lipid-lowering and hepatoprotective effect of L-theanine on NCD fed mice. Moreover, 300 mg/kg is a relatively effective dose compared to 150 and 600 mg/kg. Hence, subsequent experiments used 300 mg/kg L-theanine for HFD-induced mice.

### L-theanine alleviates nonalcoholic liver steatosis in HFD-induced mice

**Fig. 2:**
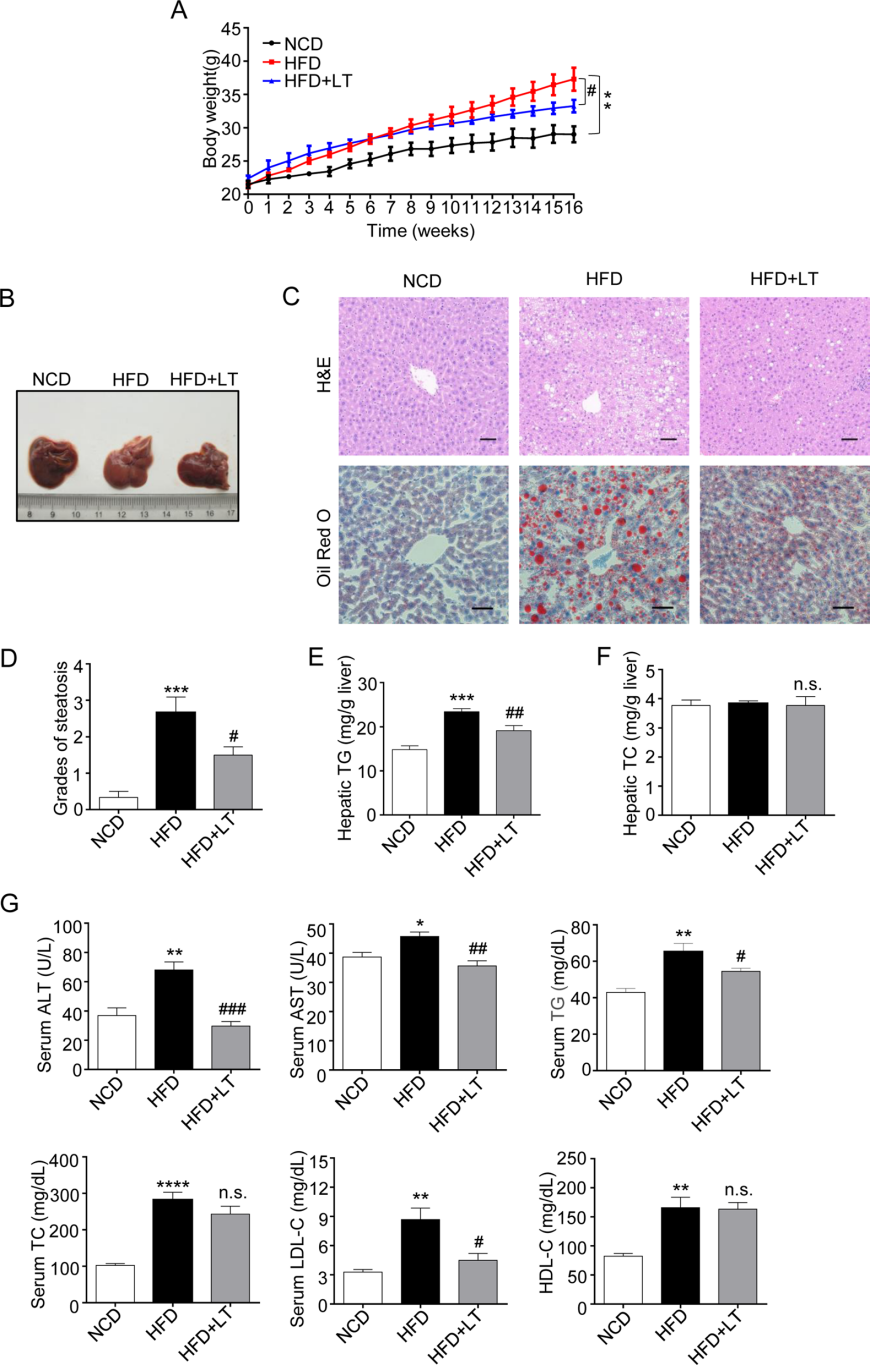
L-theanine alleviates nonalcoholic liver steatosis in HFD-induced mice. C57BL/6J mice were fed normal control diet (NCD), HFD or HFD supplemented with L-Theanine for 16 weeks, mice were gavaged with 300 mg/kg L-theanine once a day for 16 weeks, saline were gavaged as control. **A** Body weight gain curves of mice in indicated groups. **B** Macroscopic images of liver in mice. **C** Representative images of H&E and Oil Red O staining of liver tissue sections are shown. Scale bar: 50 μm (20 ×). **D** Activity score of NAFLD. Grades of steatosis are as follows: 0, no steatosis; 1, mild; 2, moderate; 3, severe; 4, very severe. Hepatic TG (**E**) and TC (**F**) contents of liver were measured by commercial kits and expressed as milligrams per gram of tissue. **G** Serum concentrations of ALT, AST, TG, TC, LDL-C and HDL-C in indicated groups. Values are expressed as mean ± SEM (n = 8). *p < 0.05, **p < 0.01, ***p < 0.001, ****p < 0.0001 versus NCD group; ^#^*p* < 0.05, ^##^*p* < 0.01, ^###^*p* < 0.001 versus HFD group, n.s.: not significant (*p* > 0.05) versus HFD group

Then, an in vivo model of nonalcoholic liver steatosis was established in HFD-induced mice. At the end of experiment, L-theanine significantly decreased body weight which was induced by HFD (Fig. 2A) (Additional file 4: Fig. S3A, B). Result also suggested that L-theanine significantly reduced the size of inguinal (Additional file 4: Fig. S3C) and epididymal (Additional file 4: Fig. S3D) adipose tissue. Furthermore, H&E-staining performed on adipose tissue sections revealed that L-theanine significantly reduced the average size of inguinal (Additional file 4: Fig. S3E, G) and epididymal (Additional file 4: Fig. S3F, H) adipocytes.

The key pathological feature of NAFLD is the accumulation of excess TG in hepatocytes, so we next investigated whether L-theanine can inhibit TG accumulation in the liver. In normal mice, the livers are dark brown–red and have a nearly normal anatomical shape, whereas the livers of HFD mice present light yellow in color and are lackluster due to the accumulation of hepatic lipids. Surprisingly, L-theanine intervention reversed these changes to some extent (Fig. 2B). Histological analysis showed increasing fat vacuoles and lipid droplets in HFD mice, but L-theanine decreased the number and size of lipid droplets (Fig. 2C). The degree of steatosis was graded using activity score of NAFLD, L-theanine treatment group had lower NAFLD activity score compared with HFD group (Fig. 2D). Further, results suggested that L-theanine decreased hepatic TG content (Fig. 2E), however had no obvious effect on hepatic TC content (Fig. 2F). These above results suggested that L-theanine attenuated nonalcoholic liver steatosis in HFD-induced mice. Intervention with L-theanine also decreased ALT, AST, TG, TC, and LDL cholesterol levels in HFD-induced mice, However, serum TC and HDL cholesterol levels were not significantly altered by L-theanine (Fig. 2G).

### L-theanine inhibits fatty acid synthesis through down-regulating FASN, ACC1 and PPARγ

**Fig. 3:**
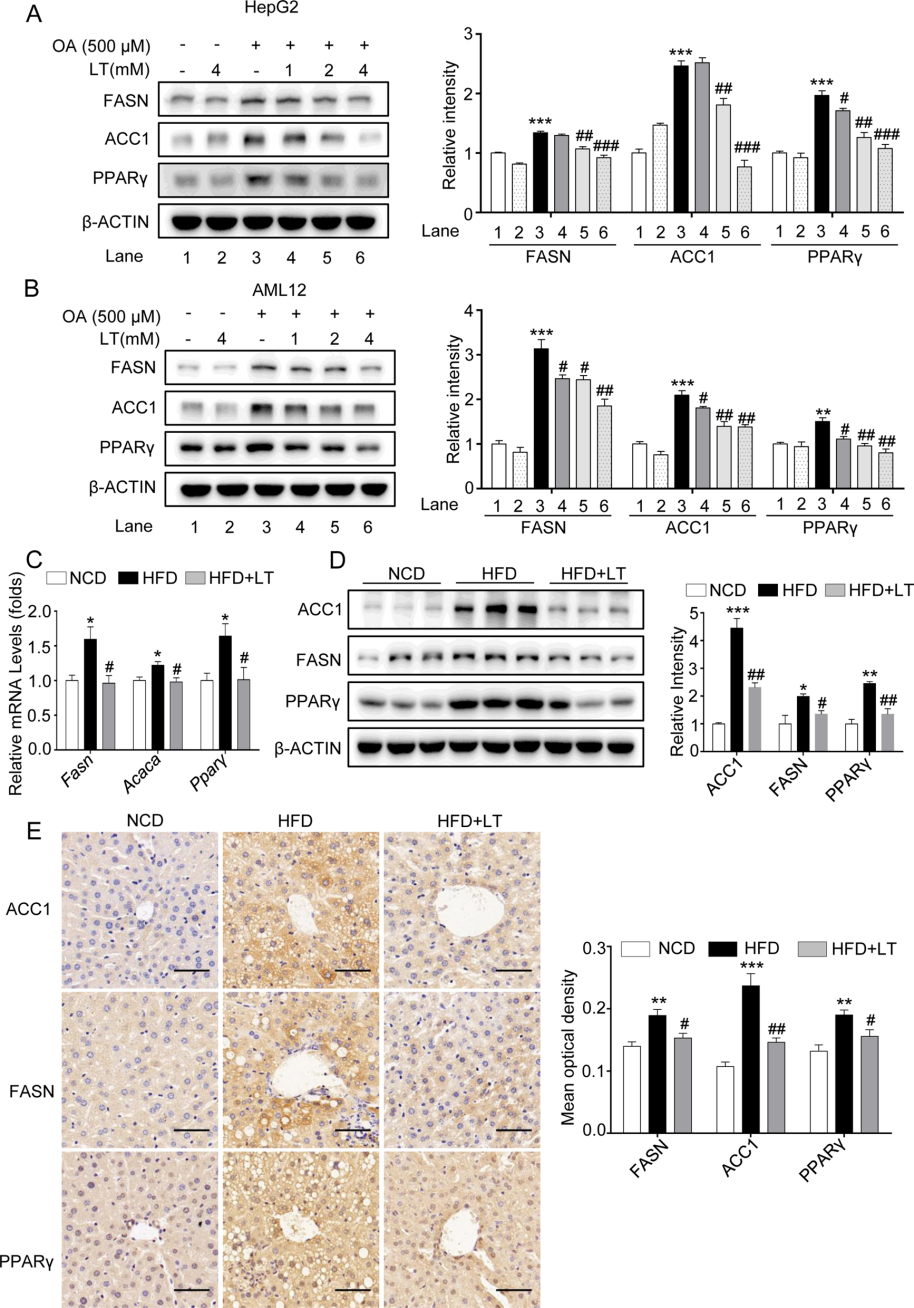
L-theanine inhibits fatty acid synthesis through down-regulating FASN, ACC1 and PPARγ. HepG2 and AML12 cells were treated with 500 μM OA for 24 h with or without pretreated L-theanine for 2 h. Western blot analysis showing protein expression of FASN, ACC1 and PPARγ in HepG2 (**A**) and AML12 (**B**) cells. C57BL/6J mice were fed normal control diet (NCD), HFD or HFD supplemented with L-Theanine for 16 weeks. **C** Real­time quantitative PCR analyses the levels of *Fasn*, *Acaca* and *Pparγ* mRNA expression in the mice liver. **D** Western blot analysis showing protein expression of FASN, ACC1 and PPARγ in mice liver. **E** Representative images of immunohistochemical staining of FASN, ACC1 and PPARγ in liver sections. Scale bar: 50 μm (40 ×). Band intensity was quantified by densitometry analysis. Values are expressed as mean ± SEM of three independent experiments. *p < 0.05, **p < 0.01, ***p < 0.001 versus control or NCD group; ^#^*p* < 0.05, ^##^*p* < 0.01, ^###^*p* < 0.001 versus OA or HFD group

To investigate the mechanisms by which L-theanine regulates fatty acid metabolism, we first looked into fatty acid synthesis pathways. Apart from rate-limiting enzymes like ACC1 and FASN, several fatty acid synthesis/lipolysis genes are regulated by peroxisome proliferator-activated receptor γ (PPARγ) in liver, and hepatocyte-specific PPARγ KO mice are protected from HFD-induced steatosis [26]. In HepG2 and AML12 cells, Western blot demonstrated that L-theanine decreased the expressions of FASN, ACC1 and PPARγ compared with OA group (Fig. 3A, B). In HFD-indued mice, L-theanine significantly decreased the mRNA and protein expressions of FASN, ACC1 and PPARγ (Fig. 3C, D), This was further validated by Immunohistochemistry (Fig. 3E). These results demonstrated that L-theanine decreased hepatic TG content through inhibiting the expressions of genes related to fatty acid synthesis.

### L-theanine inhibits fatty acid synthesis through mTOR-SREBP-1c-ACC1/FASN signaling pathway

**Fig. 4:**
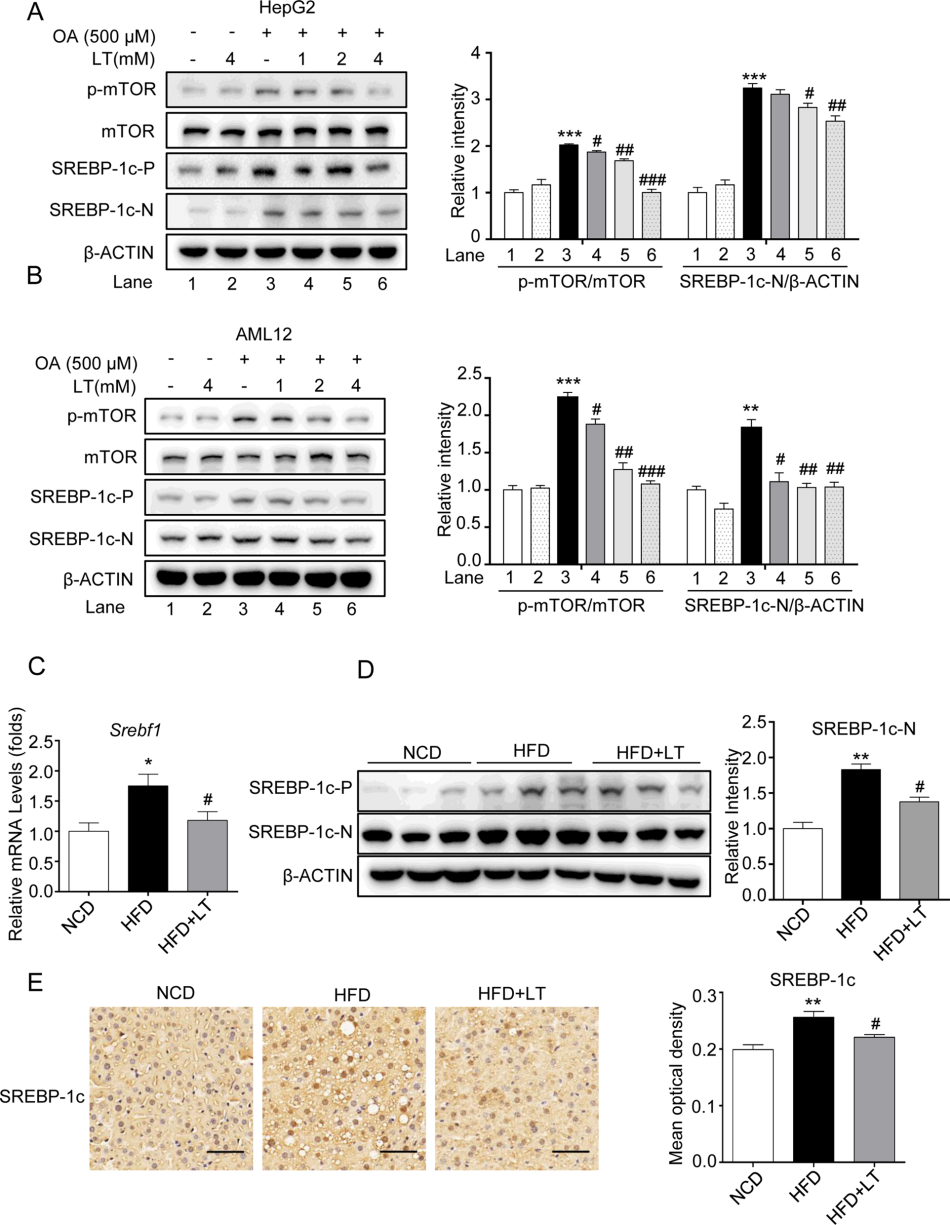
L-theanine inhibits fatty acid synthesis through mTOR-SREBP1c-ACC1/FASN signaling pathway. HepG2 and AML12 cells were treated with 500 μM OA for 24 h with or without pretreated L-theanine for 2 h. Western blot analysis showing protein expression of SREBP -1c, p-mTOR,  mTOR in HepG2 (**A**) and AML12 cells (**B**). C57BL/6J mice were fed normal control diet (NCD), HFD or HFD supplemented with L-Theanine for 16 weeks. **C** mRNA expression of *Srebf1* in mice liver. **D** Protein levels of SREBP-1c in mice liver. **E** Representative images of immunohistochemical staining of SREBP -1c in liver sections. Scale bar: 50 μm (40 ×). Band intensity was quantified by densitometry analysis. Values are expressed as mean ± SEM of three independent experiments. *p < 0.05, **p < 0.01, ***p < 0.001 versus control or NCD group; ^#^*p* < 0.05, ^##^*p* < 0.01, ^###^*p* < 0.001 versus OA or HFD group. SREBP-1c-P: precursor form of SREBP-1c; SREBP-1c-N: nuclear active form of SREBP-1c

As mentioned above, SREBP-1c is a major transcription factor that controls fatty acid synthesis through regulating the transcription of ACC1 and FASN. As the mature form of SREBP-1c is located in the nucleus, we next mainly investigated the effect of L-theanine on the nuclear active form of SREBP-1c (SREBP-1c-N). Western blot demonstrated that L-theanine decreased the expression of SREBP-1c-N compared with OA group both in HepG2 and AML12 cells (Fig. 4A, B). In HFD-induced mice, L-theanine significantly decreased the mRNA expression of *Srebf1* (Fig. 4C), Western blot was consistent with above results (Fig. 4D). The result of Immunohistochemistry also showed that L-theanine decreased the expression of SREBP-1c-N in nucleuses (Fig. 4E).

**Fig. 5:**
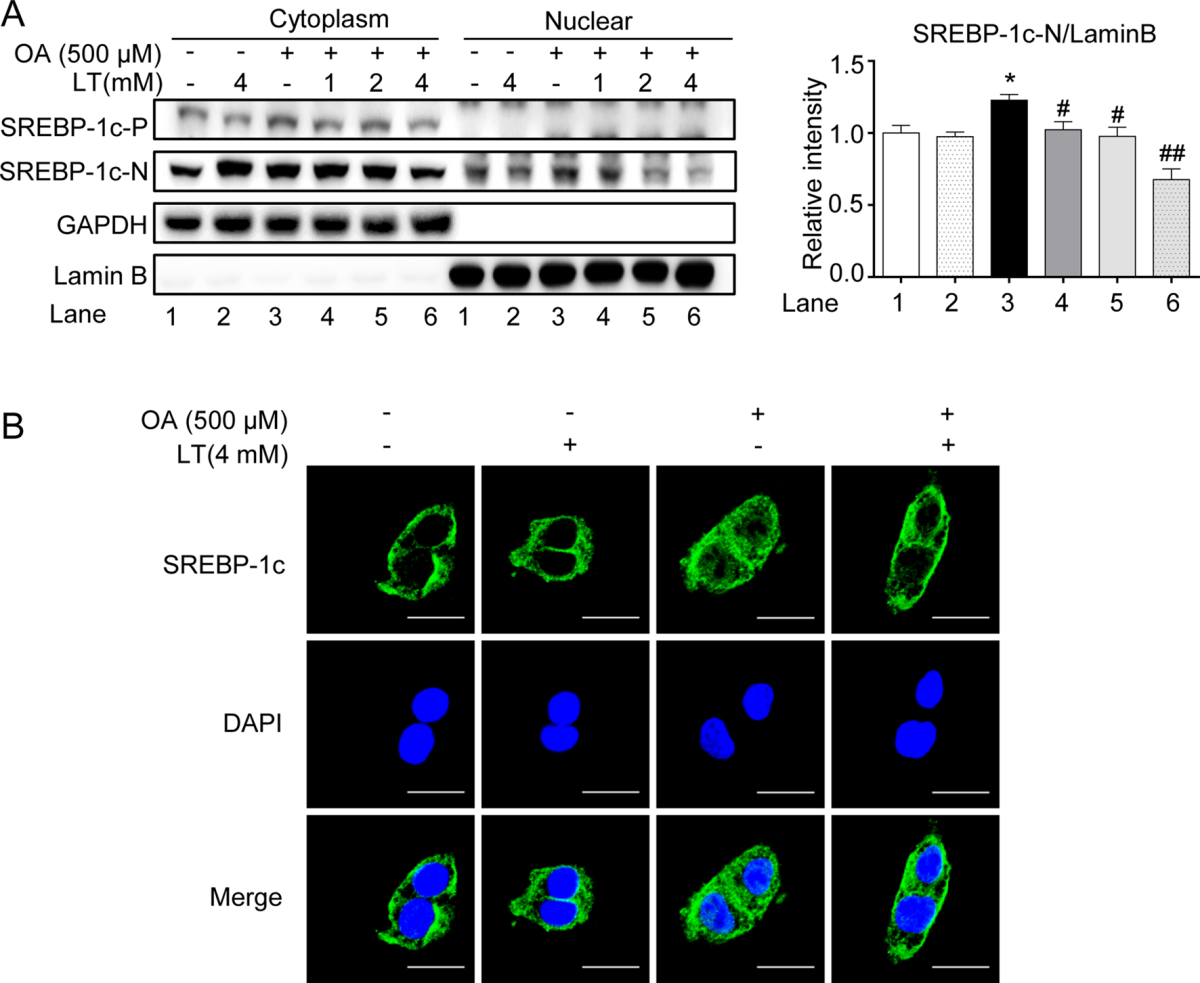
L-theanine inhibits the nucleus translocation of SREBP-1c. **A** Immunoblotting was performed to detect the level of SREBP-1c in the nucleus and cytoplasm. **B** Immunofluorescence analysis was performed to explore the effect of L-theanine (4 mM) on the nuclear translocation of SREBP-1c in HepG2 cells. Scale bar: 10 μm (60 ×). Band intensity was quantified by densitometry analysis. Values are expressed as mean ± SEM of three independent experiments. *p < 0.05 versus control group; ^#^*p* < 0.05, ^##^*p* < 0.01 versus OA group

Immunoblotting of nuclear and cytoplasmic fractions and confocal microscopy imaging were performed to detect the translocation of SREBP-1c. As immunoblotting showed, L-theanine significantly suppressed the expression of SREBP-1c-N in the nucleus which was increased in OA group (Fig. 5A). Further, confocal microscopy imaging (Fig. 5B) of SREBP-1c also showed weaker fluorescence of nucleus in L-theanine group.

As mentioned above, phosphorylation of mTOR promoted the nuclear translocation of SREBP-1c, and our results indicated that L-theanine inhibited the phosphorylation of mTOR in HepG2 and AML12 cells (Fig. 4A).

Taken together, these results confirmed that L-theanine regulated fatty acid synthesis through mTOR-SREBP-1c-ACC1/FASN pathway.

### L-theanine enhances the fatty acid β-oxidation through promoting the expressions of PPARα and CPT1A

**Fig. 6:**
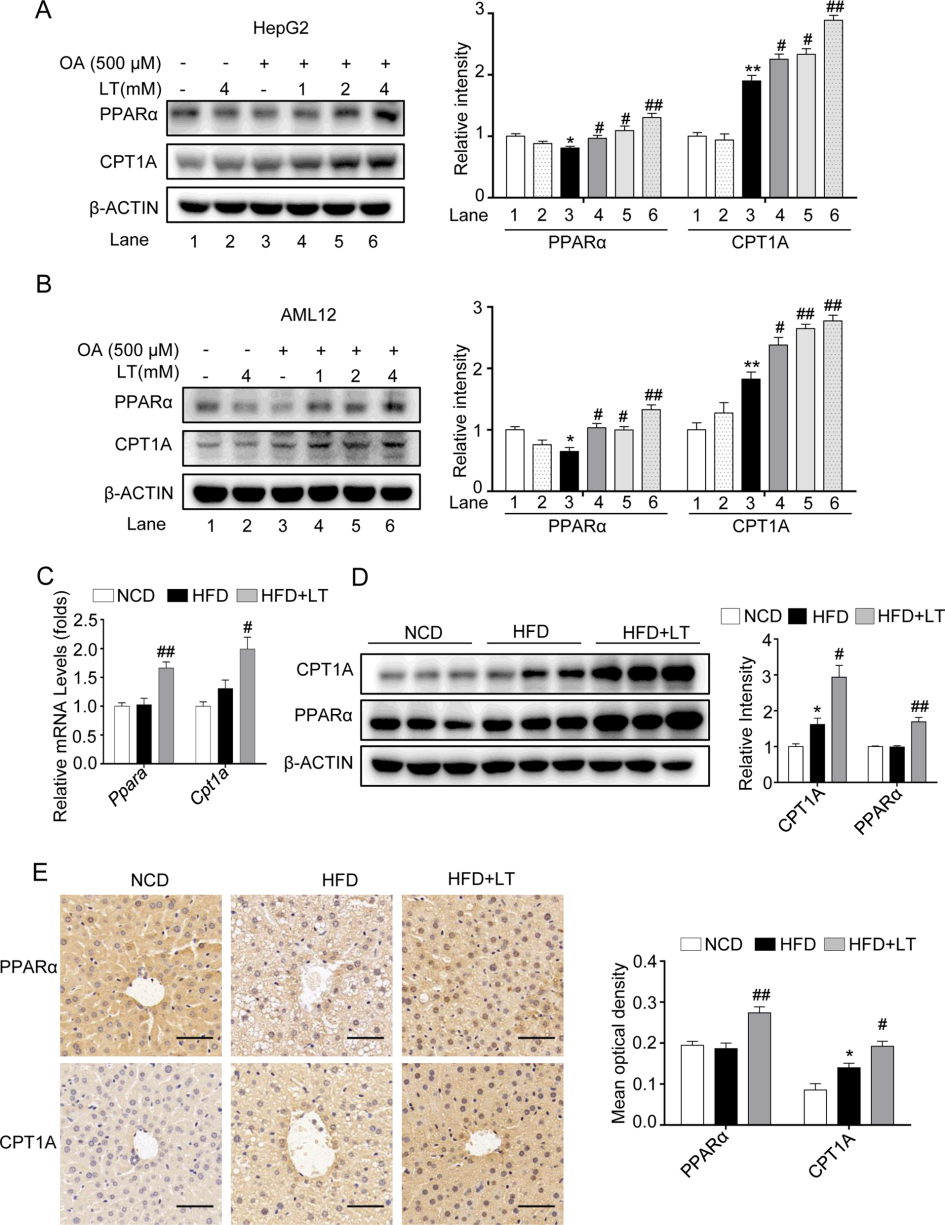
L-theanine promotes fatty acid β oxidation through promoting the expressions of PPARα and CPT1A. HepG2 and AML12 cells were treated with 500 μM OA for 24 h with or without pretreated L-theanine for 2 h. Western blot analysis showing protein expression of PPARα and CPT1A in HepG2 (**A**) and AML12 (**B**) cells. C57BL/6J mice were fed normal control diet (NCD), HFD or HFD supplemented with L-Theanine for 16 weeks. **C** mRNA expression of *Ppara* and *Cpt1a* in mice liver. **D** Protein levels of PPARα and CPT1A in mice liver. **E** Representative images of immunohistochemical staining of PPARα and CPT1A in liver sections. Scale bar:50 μm (40 ×). Band intensity was quantified by densitometry analysis. Values are expressed as mean ± SEM of three independent experiments. *p < 0.05, **p < 0.01 versus control or NCD group; ^#^*p* < 0.05, ^##^*p* < 0.01 versus OA or HFD group

Apart from the abnormality of fatty acid synthesis, lipid accumulation in liver is also related to β-oxidation. Next, we investigated the effect of L-theanine on fatty acid β-oxidation. In HepG2 and AML12 cells, L-theanine significantly promoted the protein expressions of peroxisome proliferator-activated receptor α (PPARα) and carnitine palmitoyltransferase-1 A (CPT1A) (Fig. 6A, B). In mice, L-theanine promoted the mRNA and protein expressions of PPARα and CPT1A compared with HFD group (Fig. 6C, D). Immunohistochemistry showed that PPARα had a higher expression in nuclear in L-theanine group compared with HFD group (Fig. 6E). These results indicated that L-theanine enhanced fatty acid β-oxidation by promoting the translocation of PPARα.

### L-theanine regulates hepatocyte lipid metabolic pathways via the CaMKKβ-AMPK signaling pathway

**Fig. 7:**
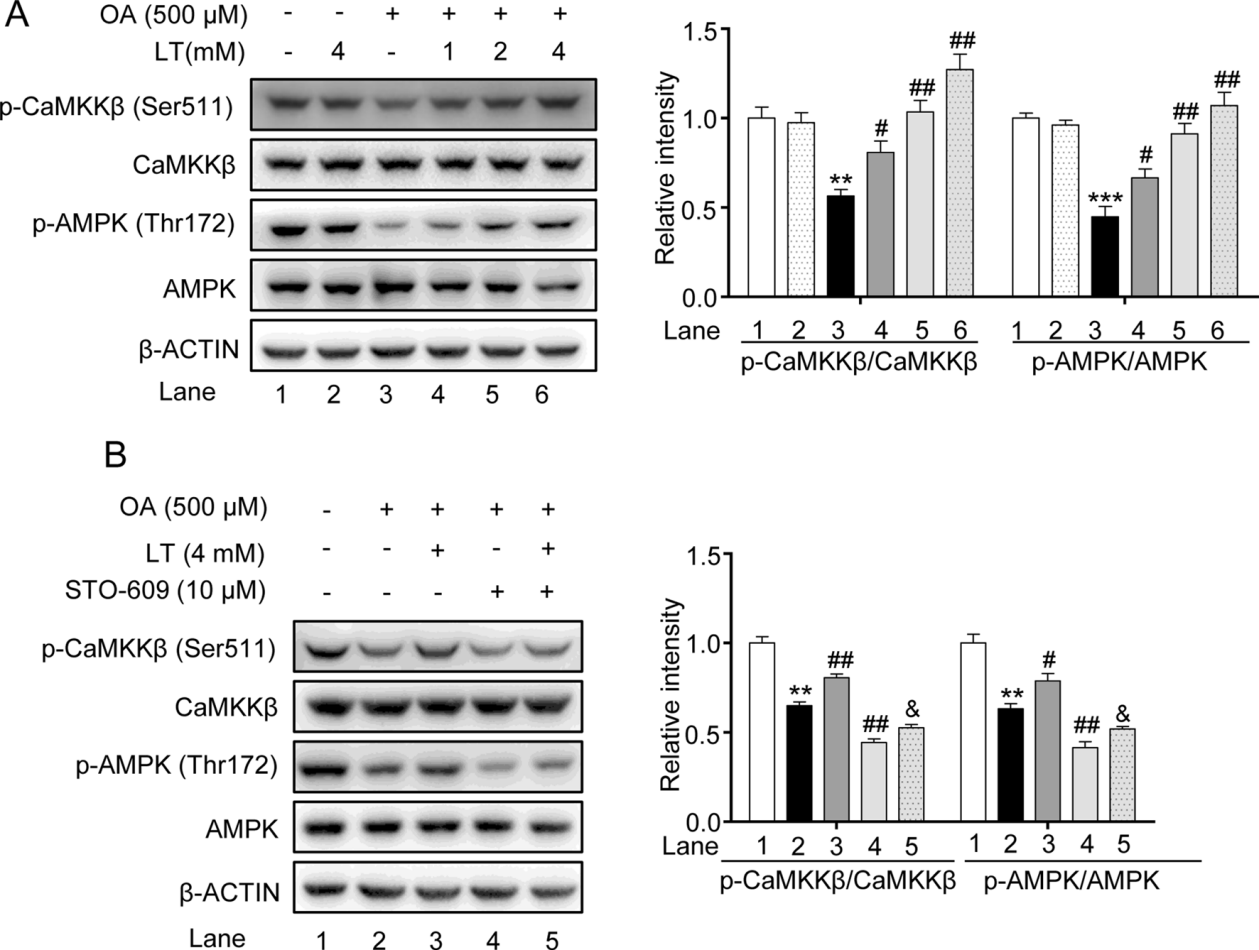

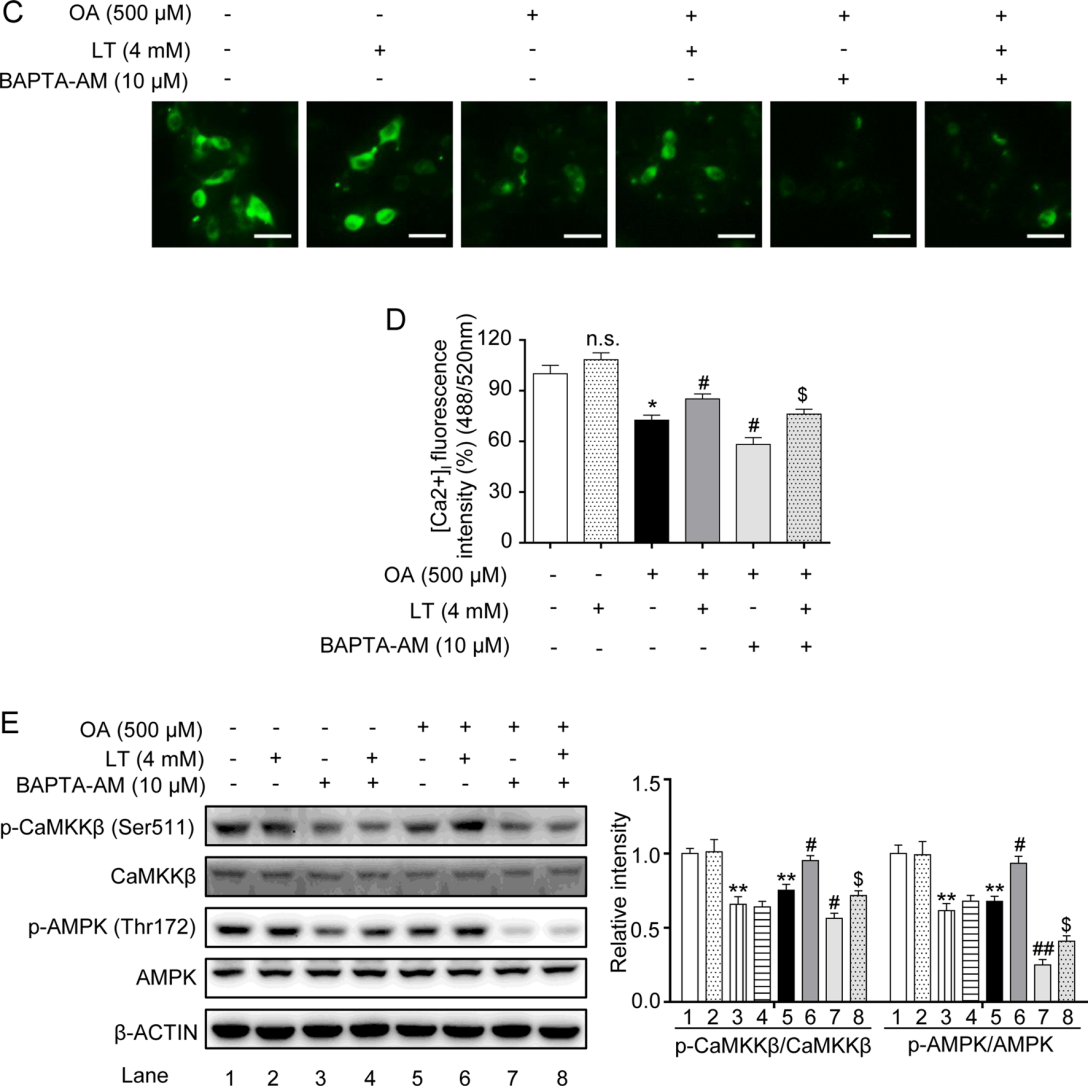
L-theanine regulates hepatocyte lipid metabolic pathways via the CaMKKβ-AMPK signaling pathway. HepG2 cells were treated with 500 μM OA for 24 h with or without pretreated L-theanine for 2 h. **A** Western blot analysis showing protein expression of p-CaMKKβ and p-AMPK in HepG2 cells. HepG2 cells were pretreated with 10 μM STO-609 for 2 h, then added L-theanine co-incubated for 2 h, finally, OA were added co-incubated for 24 h. **B** Abrogating effect of CaMKKβ inhibitor on L-theanine-facilitated phosphorylation of CaMKKβ and AMPKα in HepG2 hepatocytes. HepG2 cells were pretreated with 10 μM BAPTA-AM for 1 h, then added L-theanine co-incubated for 2 h, finally, OA were added co-incubated for 24 h. **C** Intracellular Ca^2+^ levels were assessed in HepG2 cells with Fluo-4 AM using a fluorescence microscope. Scale bar: 20 μm (20 ×). **D** [Ca^2+^]_i_ was detected by a multifunctional microplate reader using Fluo-4 AM. under the condition of 488 nm excitation wavelength and 520 nm emission wavelength. **E** Western blots of indicated proteins in HepG2 cells treated with an intracellular Ca^2+^ chelator BAPTA-AM. Band intensity was quantified by densitometry analysis. Values are expressed as mean ± SEM of three independent experiments. *p < 0.05, **p < 0.01, ***p < 0.001, versus control group; ^#^*p* < 0.05, ^##^*p* < 0.01, versus OA group; ^&^*p* < 0.05 versus OA + STO-609 group, ^$^*p* < 0.05 versus OA + BAPTA-AM group. n.s.: not significant (*p* > 0.05) versus control group

As a cellular energy sensor, AMPK plays an essential role in hepatic lipid metabolism. We next invested the effect of L-theanine on AMPK in OA induced HepG2 cells. Western blot analysis showed that L-theanine significantly activated AMPK at threonine 172 (Fig. 7A). AMPK is activated by two upstream kinases, LKB1 and CaMKKβ. A previous study demonstrated that L-theanine can activate AMPK through LKB1 in normal rats [15]. In the present study, we invested whether CaMKKβ was involved in the activation of AMPK, as the result showed, L-theanine activated the phosphorylation of CaMKKβ in a dose dependent manner (Fig. 7A). STO-609 (a selective CaMKKβ inhibitor) significantly reduced the phosphorylation of CaMKKβ and AMPK in OA group, and the reversed results were obtained after L-theanine treatment (Fig. 7B). Previous study demonstrated that CaMKKβ is activated by Ca^2+^/calmodulin, CaMKKβ can activate AMPK under conditions in which intracellular Ca^2+^ is increased [27]. Next, we detected the effect of L-theanine on the intracellular Ca^2+^ concentration. As shown in Fig. 7C, compared with OA group, the fluorescence intensity was significantly higher in OA plus L-theanine group. BAPTA-AM, an intracellular Ca^2+^ chelator, significantly reduced the fluorescence intensity, and L-theanine reversed this trend to some extent. Intracellular Ca^2+^ levels detected by a multifunctional microplate reader were consistent with the above result (Fig. 7D). Western blot analysis showed that phosphorylation of CaMKKβ and AMPK was inhibited by BAPTA-AM, and L-theanine treatment significantly reversed the inhibition (Fig. 7E). These data indicated that L-theanine also can activate AMPK through Ca^2+^-CaMKKβ.

## Discussion

Previous studies have reported that L-theanine can inhibit obesity in mice [13], regulate lipid metabolism in normal rats [15], reduce the risk of T2DM development in human [12]. Recent study indicated that L-theanine can activate the browning of white adipose tissue [16] and modulation of gut microbiota [17] in obesity mice. As mouse with high fat diet (60 kcal% Fat) for 16 weeks may not reach the severity of hepatic fibrosis of full-blown NASH [28], we mainly studied the effects of L-theanine on simple steatosis of liver in the current study. We found that L-theanine can ameliorates nonalcoholic hepatic steatosis by regulating hepatocyte lipid metabolic pathways via the CaMKKβ-AMPK signaling pathway.

Long-term exogenous high-fat diet and overnutrition is the main factor for the development of NAFLD in the clinical condition [29]. Therefore, HFD-induced animal model is used to mimic the pathophysiology of nonalcoholic hepatic steatosis in human. Our results showed that, compared with HFD group, the body weight gain, severity of hepatic steatosis, serum levels of ALT, AST, TG and LDL-C were decreased in L-theanine group. Our results were consistent with previous reports [16, 17]. Result also showed that L-theanine had no effect on serum levels of HDL-c and TC in HFD-induced mice. As HDL plays an important role in reverse cholesterol transport (RCT) [30], our results demonstrated that L-theanine may have no effect on RCT under the condition of NAFLD.

The key pathological feature of hepatic steatosis is the accumulation of excess TG in hepatocytes, and studies had shown that a large part of TG in liver with NAFLD was derived from fatty acid synthesis [31]. Previous studies have shown that L-theanine can decrease the expression of FASN and ACC1 in the liver of normal rats [15], and the mRNA expression in HFD-induced liver [17], Our results showed that L-theanine effectively decreased the mRNA and protein expression of FASN, ACC1 and PPARγ in steatosis liver in vitro and in vivo.

FASN and ACC1 are transcriptionally controlled by various transcriptional regulators, especially SREBP-1c [19]. SREBP-1c is synthesized as an inactive precursor in cytoplasm, after the cleavage into the mature form, it shuttles to the nucleus and induces the expression of FASN and ACC1 [18]. In the current study, our data indicated that L-theanine can inhibit the expression of SREBP-1c-N in hepatocytes. Furthermore, we found that L-theanine inhibited the nucleus translocation of SREBP-1c.

As a key regulator of cell metabolism and growth in response to nutritional and hormonal stimuli, mTOR deregulation has been implicated in many disease states, including diabetes, obesity and NAFLD. Studies found that mTOR promote the trafficking, processing, and transcription of SREBPs [32]. Previous study showed that mTOR can active SREBP-1c in an S6K1-dependent manner [33], and mTOR also can regulate SREBP-1c through regulating the localization of lipin 1 [34]. Research also showed that mTOR can activate SREBP-1c maturation through inhibiting CRTC2 activation, a coactivator that have inhibitory effect on SREBP-1c maturation [35]. In the current study, L-theanine treatment can inhibit the phosphorylation of mTOR which was up-regulated in OA induced hepatocytes. Our data suggested that L-theanine can inhibit the maturation of SREBP-1c through inhibiting phosphorylation of mTOR.

Previous studies showed that the underlying mechanisms of L-theanine action on metabolic disorders appeared to be primarily mediated by the AMPK pathway. Lin et al. showed that L-theanine can regulate lipid metabolism through AMPK in the liver of normal rats [15]. Peng et al. showed that L-theanine can regulate a thermogenic program in white adipose tissue through AMPK [16]. Enhancing the activity of AMPK can prevent and improve nonalcoholic liver steatosis to some extent [36]. As an upstream kinase of mTOR, activation of AMPK can inhibit fatty acid synthesis through down-regulating the activity of mTOR [37]. Our results suggested that L-theanine inhibited fatty acid synthesis through AMPK-mTOR-SREBP-1c-FASN/ACC1 signaling pathway.

Activation of AMPK also can increase fatty acid β-oxidation [38]. Overexpression of AMPKα1 in the liver increase the expression of CPT1a, which is the rate-limiting enzyme in fatty acid β-oxidation. Previous studies have shown that L-theanine can increase the mRNA expression of *Cpt1a* in the liver of normal rats [15] or HFD-induced mice [17]. Our results indicated that L-theanine can promote fatty acid β-oxidation through up-regulating the mRNA and protein expression of PPARα and CPT1A in vivo and in vitro [39]. Our results suggested that L-theanine can activate AMPK at Thr 172 on the AMPKα subunit.

To date, two AMPK upstream kinases have been characterized, the first is the tumor suppressor liver kinase B1 (LKB1) [40] and the second is Ca^2+^/Calmodulin-dependent protein kinase kinase β (CaMKKβ) [41]. Previous study demonstrated that L-theanine can activate AMPK via its upstream kinases LKB1 in normal rat liver [15]. In the current study we invested another AMPK upstream kinase CaMKKβ. Our results demonstrated L-theanine can affect the level of intracellular Ca^2+^ and active AMPK through CaMKKβ. Studies showed that LKB1 can response to a change in the AMP:ATP ratio, thus actives AMPK via AMP, and CaMKKβ activate AMPK mainly through Ca^2+^, however, the increase of Ca^2+^ would also change the AMP:ATP ratio, and vice versa [42]. So, in many cases, the activation of AMPK is the consequence of both LKB1 and CaMKKβ [27]. Thus, the activation of AMPK mediated by L-theanine may via both LKB1 and CaMKKβ. Our data suggested that the effects of L-theanine may be used to develop a novel regent for the management of nonalcoholic liver steatosis.

## Conclusions

**Fig. 8:**
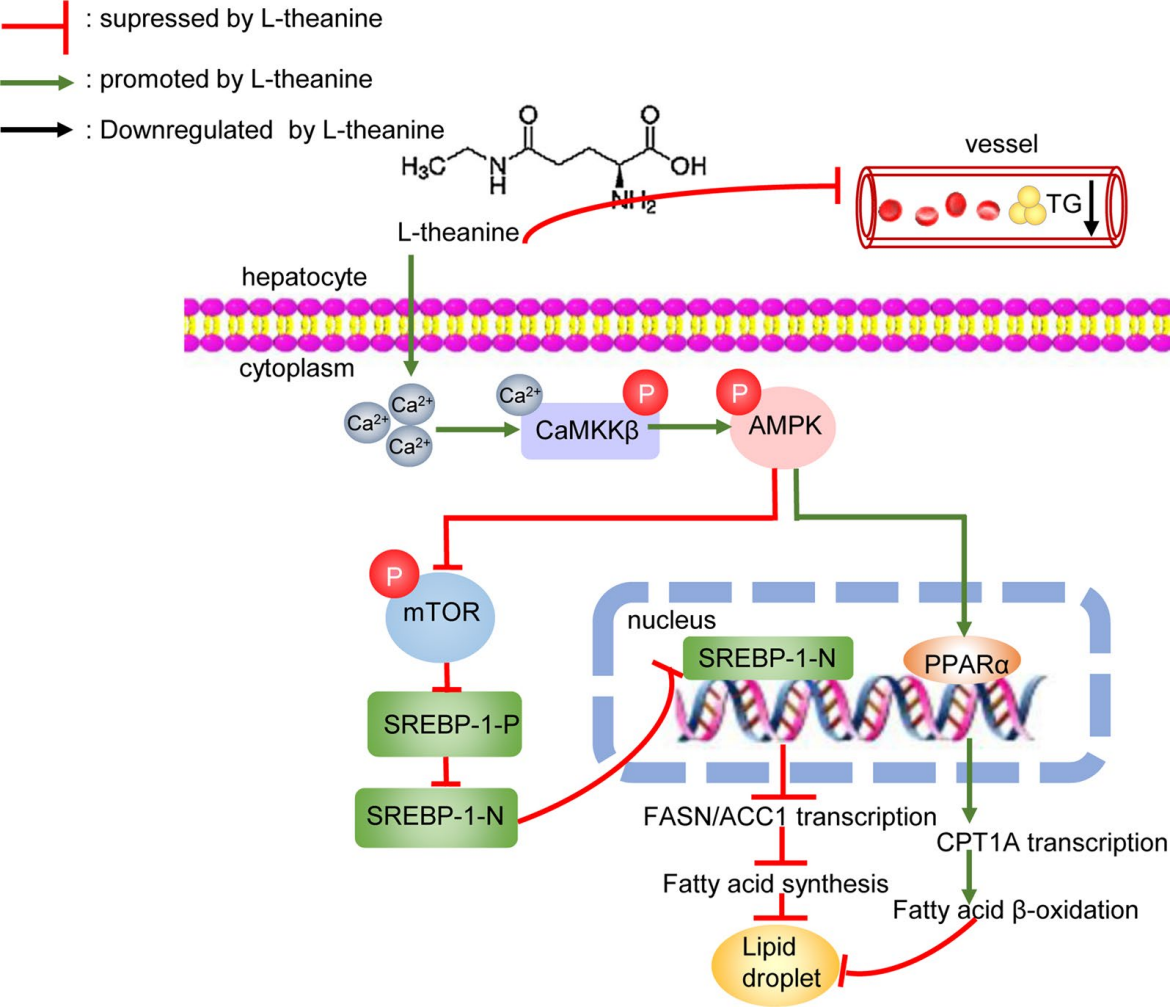
Schematic of the role of L-theanine in protecting against nonalcoholic hepatic steatosis

In summary, our experimental data indicated that L-theanine can protect against nonalcoholic hepatic steatosis in vitro and in vivo*.* Our mechanic studies indicated that L-theanine can regulate lipid metabolism of nonalcoholic fatty liver through activating Ca^2+^-CaMKKβ-AMPK signaling pathway, inhibit the expression of ACC1, FASN and PPARγ which were related to fatty acid synthesis. At the same time, activation CaMKKβ-AMPK promoted the expression of PPARα and CPT1A which were related to fatty acid β-oxidation (Fig. 8). We explained that L-theanine can affect the level of intracellular Ca^2+^ and active AMPK through CaMKKβ.

We explained a specific mechanism by which theanine alleviated nonalcoholic hepatic steatosis, and proved that L-theanine may be a novel regent for the treatment of nonalcoholic hepatic steatosis.

## Supplementary Information

**Additional file 1: Table S1**. Primers for Real-Time PCR detection.

**Additional file 2: Fig. S1**. Effects of L-theanine and OA on survival rate of HepG2 and AML12 cells. The effects of different concentrations of L-theanine on survival rate of HepG2 (A) and AML12 (B) cells. Effects of different concentrations of OA on survival rate of HepG2 (C) and AML12 (D) cells. Values are expressed as mean ± SEM of three independent experiments. **p < 0.01, ***p < 0.001, ****p < 0.0001 vs control group. n.s.: not significant (p > 0.05) vs control group.

**Additional file 3: Fig. S2**. The effects of L-theanine on the metabolism of normal mice. Normal fed mice (NCD) were given different concentrations of L-theanine (150 mg/kg, 300 mg/kg, 600 mg/kg) by gavage, and NCD group was given 0.9% normal saline by gavage as control. (A) The body weight growth curve of mice in different treatment groups during 1-16 weeks. (B) Body weight gain of mice in different treatment groups at 16th week. (C) Serum level of ALT, AST, TG, TC, LDL-C and HDL-C in mice of different treatment groups. Values are expressed as mean ± SEM (n = 6). *p<0.05 vs NCD group, n.s.: not significant (p > 0.05) vs NCD group.

**Additional file 4: Fig. S3**. Effects of L-theanine on the body weight and adipose tissue size in HFD-induced mice. (A) The appearance of mice. (B) The body weight gain of mice. The size of inguinal (C) and epididymal (D) adipose tissue. Representative images of H&E-staining and quantification of inguinal (E, G) and epididymal (F, H) adipose tissue sections. Scale bar:50 μm (20 × ). Values are expressed as mean ± SEM (n = 8). **p < 0.01, ***p < 0.001 vs NCD group; #p < 0.05, ##p < 0.01 vs HFD group.

## Data Availability

The datasets used and/or analysed during the current study are available from the corresponding author on reasonable request.

## Abbreviations

NAFLD: Nonalcoholic fatty liver disease
TG: Triglycerides
TC: Total cholesterol
ALT: Alanine aminotransferase
AST: Aspartate aminotransferase
HDL-C: HDL cholesterol
LDL-C: LDL cholesterol
HFD: High-fat-diet
NCD: Normal control diet
OA: Oleic acid
SREBP-1c: Sterol regulatory element-binding protein-1c
SREBP-1c-P: Precursor form of SREBP-1c
SREBP-1c-N: Nuclear active form of SREBP-1c
FASN: Fatty acid synthase
ACC1: Acetyl CoA carboxylase
PPARγ: Peroxisome proliferator-activated receptor γ
PPARα: Peroxisome proliferator-activated receptor α
CPT1A: Carnitine palmitoyltransferase-1 A
AMPK: AMP-activated protein kinase
CaMKKβ: Calmodulin-dependent protein kinase kinase-β
