# Supplementary material for: L-theanine prevents progression of nonalcoholic hepatic steatosis by regulating hepatocyte lipid metabolic pathways via the CaMKKβ-AMPK signaling pathway
Source: Nutr Metab (Lond). 2022 Apr 15;19:29. doi: 10.1186/s12986-022-00664-6 (PMC9013079; doi:10.1186/s12986-022-00664-6)
Supplement: Supplementary file 1 — Additional file 1: Table S1. Primers for Real-Time PCR detection. [file 12986_2022_664_MOESM1_ESM.docx]

Table S1. Primers for Real-Time PCR detection

| Gene | Sequence 5’→3’ |
| --- | --- |
| Mouse *Srebf1* | F：CAAGGCCATCGACTACATCCG  R：CACCACTTCGGGTTTCATGC |
| Mouse *Fasn* | F：CCGGAGTCGCTTGAGTATATTG  R：TTGTGGAAGTGCAGGTTAGG |
| Mouse *Acaca* | F：CTTCCTGACAAACGAGTCTGG  R：CTGCCGAAACATCTCTGGGA |
| Mouse *Ppar**γ* | F：CTCCAAGAATACCAAAGTGCGA  R：GCCTGATGCTTTATCCCCACA |
| Mouse *Ppara* | F：AGAACCTGAGGAAGCCGTTC  R：TTAAGCACGTGCACAATCCC |
| Mouse *Cpt1a* | F：TGACGGCTATGGTGTTTCCT  R：TTTTGGAATTGGCGGTGAGG |
| Mouse *Actb* | F：GTGACGTTGACATCCGTAAAGA  R：GCCGGACTCATCGTACTCC |
