# Supplementary figures and images for: L-theanine prevents progression of nonalcoholic hepatic steatosis by regulating hepatocyte lipid metabolic pathways via the CaMKKβ-AMPK signaling pathway
Source: Nutr Metab (Lond). 2022 Apr 15;19:29. doi: 10.1186/s12986-022-00664-6 (PMC9013079; doi:10.1186/s12986-022-00664-6)

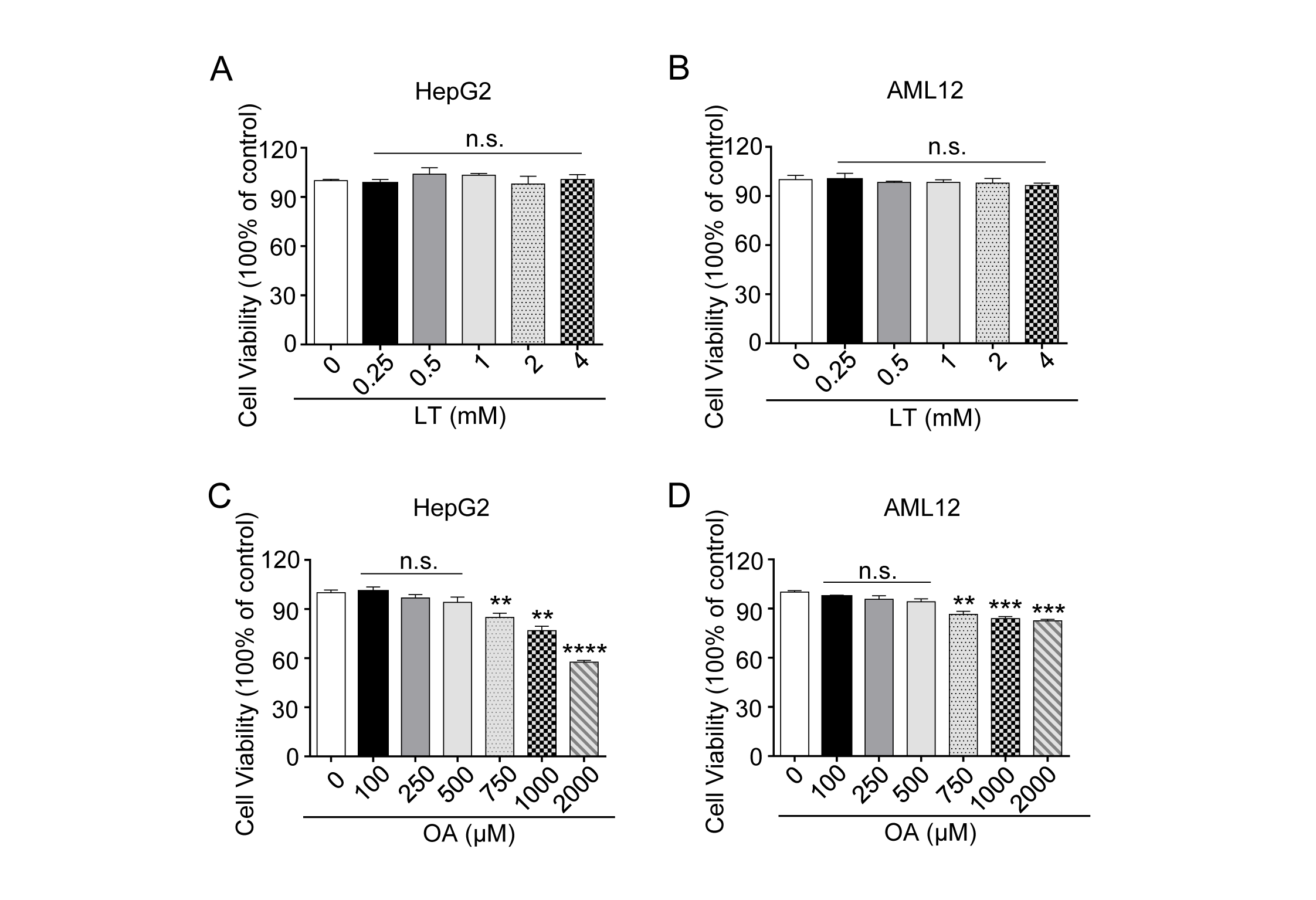

Supplement: Supplementary file 2 — Additional file 2: Fig. S1. Effects of L-theanine and OA on survival rate of HepG2 and AML12 cells. The effects of different concentrations of L-theanine on survival rate of HepG2 (A) and AML12 (B) cells. Effects of different concentrations of OA on survival rate of HepG2 (C) and AML12 (D) cells. Values are expressed as mean ± SEM of three independent experiments. **p < 0.01, ***p < 0.001, ****p < 0.0001 vs control group. n.s.: not significant (p > 0.05) vs control group. [file 12986_2022_664_MOESM2_ESM.tif]

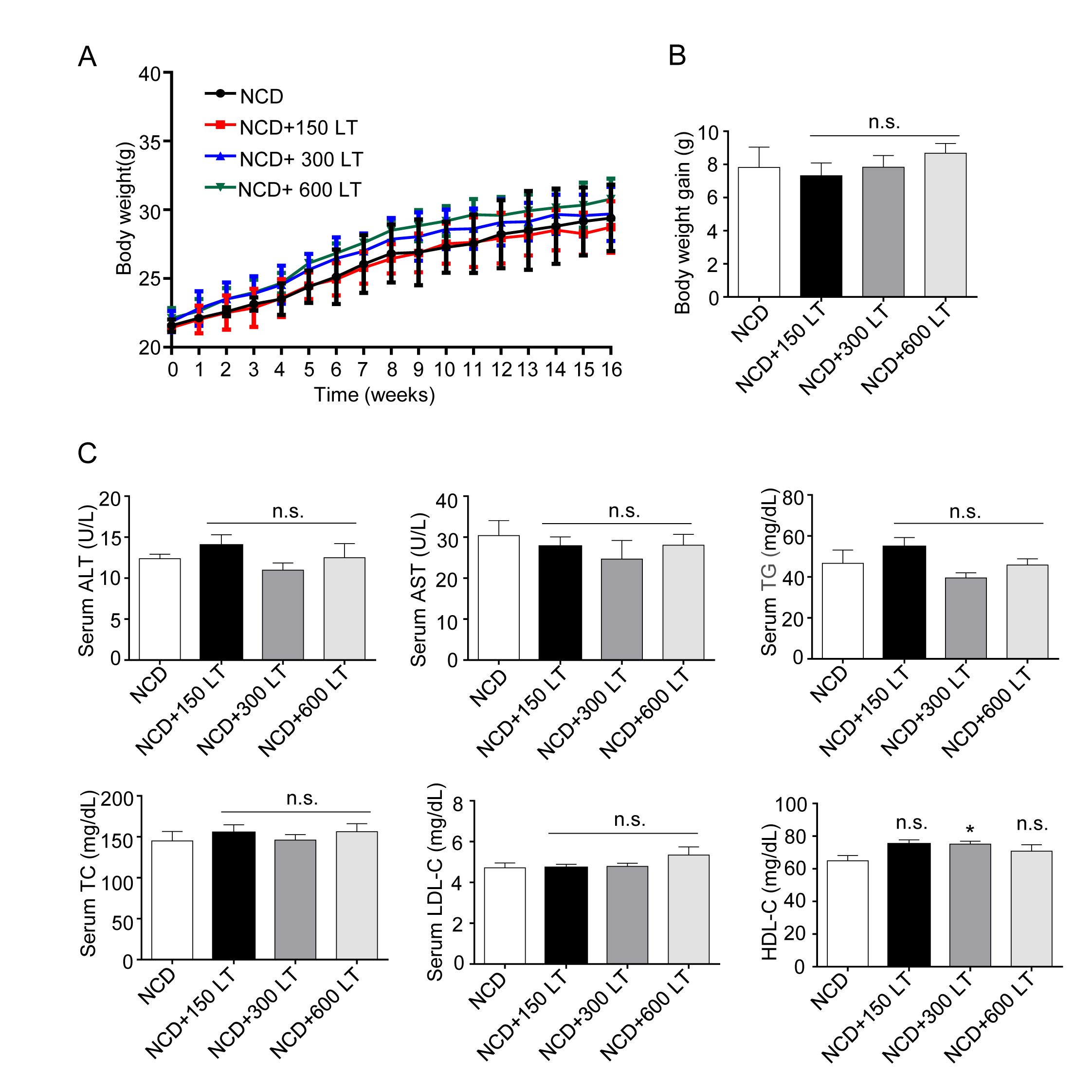

Supplement: Supplementary file 3 — Additional file 3: Fig. S2. The effects of L-theanine on the metabolism of normal mice. Normal fed mice (NCD) were given different concentrations of L-theanine (150 mg/kg, 300 mg/kg, 600 mg/kg) by gavage, and NCD group was given 0.9% normal saline by gavage as control. (A) The body weight growth curve of mice in different treatment groups during 1-16 weeks. (B) Body weight gain of mice in different treatment groups at 16th week. (C) Serum level of ALT, AST, TG, TC, LDL-C and HDL-C in mice of different treatment groups. Values are expressed as mean ± SEM (n = 6). *p<0.05 vs NCD group, n.s.: not significant (p > 0.05) vs NCD group. [file 12986_2022_664_MOESM3_ESM.tif]

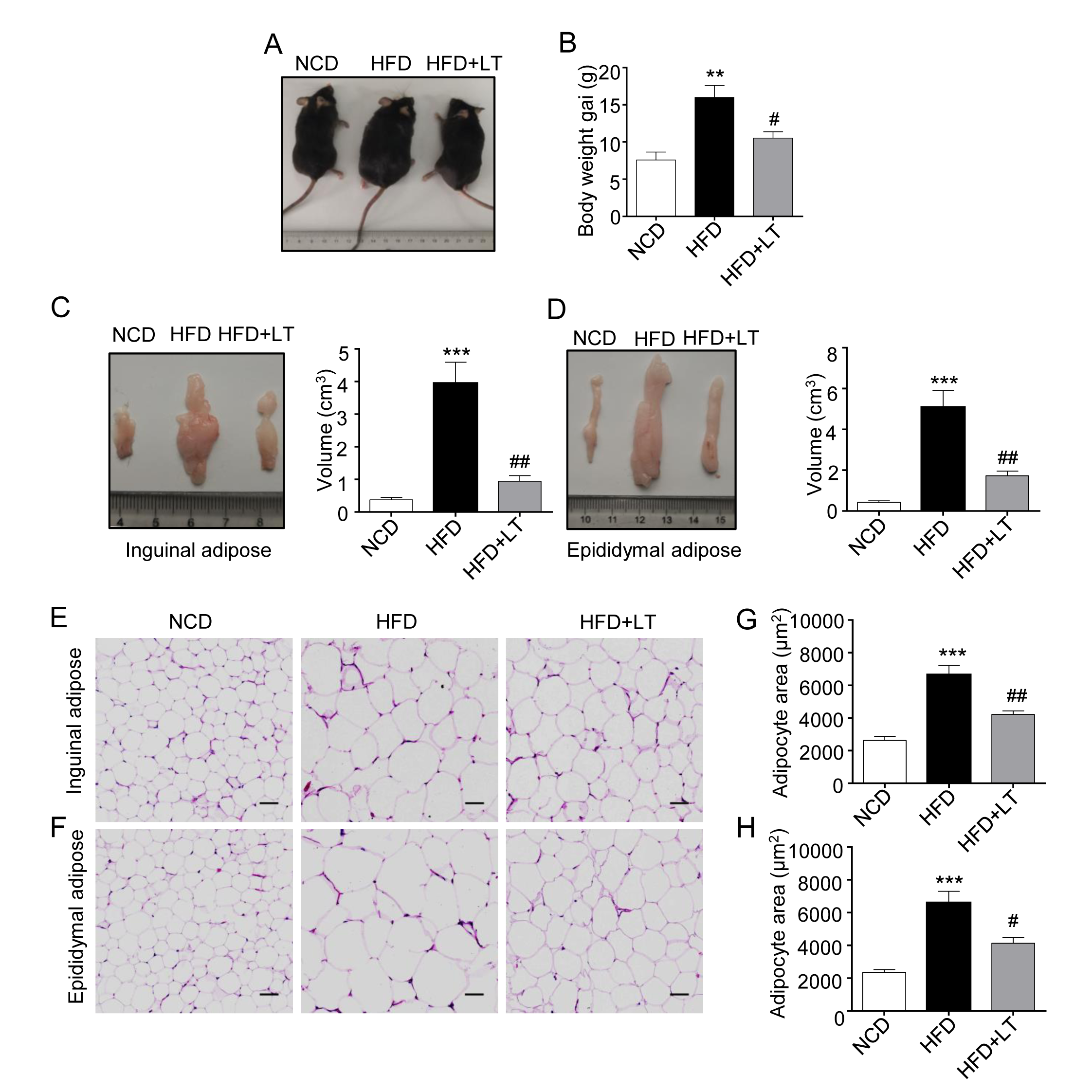

Supplement: Supplementary file 4 — Additional file 4: Fig. S3. Effects of L-theanine on the body weight and adipose tissue size in HFD-induced mice. (A) The appearance of mice. (B) The body weight gain of mice. The size of inguinal (C) and epididymal (D) adipose tissue. Representative images of H&E-staining and quantification of inguinal (E, G) and epididymal (F, H) adipose tissue sections. Scale bar:50 μm (20 × ). Values are expressed as mean ± SEM (n = 8). **p < 0.01, ***p < 0.001 vs NCD group; #p < 0.05, ##p < 0.01 vs HFD group. [file 12986_2022_664_MOESM4_ESM.tif]
